# Advancing Implementation of Evidence-Based Public Health in China: An Assessment of the Current Situation and Suggestions for Developing Regions

**DOI:** 10.1155/2016/2694030

**Published:** 2016-08-14

**Authors:** Jianwei Shi, Chenghua Jiang, Duxun Tan, Dehua Yu, Yuan Lu, Pengfei Sun, Ying Pan, Hanzhi Zhang, Zhaoxin Wang, Beilei Yang

**Affiliations:** ^1^Yangpu Hospital, Tongji University School of Medicine, Shanghai 200090, China; ^2^Tongji University School of Medicine, Shanghai 200092, China; ^3^The Fifth Affiliated Hospital of Southern Medical University, Guangzhou 510900, China; ^4^Tongji University College of Economics and Management, Shanghai 200092, China

## Abstract

*Objective*. Existing research shows a serious scarcity of EBPH practice in China and other developing regions; as an exploratory study, this study aimed to assess the current EBPH implementation status in Shanghai of China qualitatively.* Methods*. Using semistructured key informant interviews, we examined the status of and impediments to the lagging EBPH in China. Data were analyzed based on the Consolidated Framework for Implementation Research (CFIR).* Results*. Chinese public health practitioners knew more about evidence-based medicine but less about EBPH. The situation was worse in community healthcare centers. Participants perceived that evidence sources were limited and the quality of evidence was low. Concerning the inner setting factors, the structural characteristics, networks and communications, implementation climate, and leadership engagement were confronted with many problems. Among the outer setting factors, external government policies and incentives and low patient compliance were the key problems. Additionally, public health practitioners in Shanghai lacked sufficient awareness of EBPH. Furthermore, the current project-based EBPH lacks a systematic implementation system.* Conclusions*. Existing practical perspectives on EBPH indicate a lag in the advocacy of this new ideology in China. It would be advisable for healthcare institutions to take the initiative to explore feasible and multiple methods of EBPH promotion.

## 1. Introduction

### 1.1. Evidence-Based Public Health as a Booster to Public Health Promotion

Evidence-based public health (EBPH), which was formally proposed in the US approximately two decades ago, has gained widespread academic attention and obtained fast progress in high-income countries, especially since the 2000s [1]. Although there is not yet a generic definition, there appears to be a consensus among scholars and practitioners that EBPH is a combination of scientific evidence and values, resources, and context that should be used to inform public health decisions and improve the health of populations [1–4]. Over the past several years, many efforts have been devoted to developing evidence-based decision-making in public health fields in developed countries. The Cochrane Collaboration in many developed countries and the national guidelines of the “Community Guide” and “Cancer Control P.L.A.N.E.T.” in the US provide good approaches to planning and developing evidence-based programs and policies [5–7]. Under these circumstances, the use of evidence-based practices in public health has exerted numerous direct and indirect benefits, including access to more and higher-quality information on what works, a higher likelihood of successful programs and policies being implemented, greater workforce productivity, and more efficient use of public and private resources [2, 8, 9].

However, existing knowledge shows that EBPH has not achieved systematic dissemination. Studies have revealed that implementation or translation of evidence-based approaches in public health always encounters complex barriers, including personal and institutional factors and political environment and deficits in information systems, resources, and leadership [10, 11]. Additionally, lack of time and incentives, inadequate connections between research and practice, and the absence of cultural and managerial support are among the most commonly cited barriers [12–14].

### 1.2. Evidence-Based Public Health in the Context of Public Health Reform in China

Despite the growing body of research on EBPH implementation and promotion in high-income countries, there is a lack of studies that assess status of EBPH and its dissemination in China and other developing regions [15, 16]. To date, EBPH is still in its early stages in China. In 2003, the outbreak of SARS initiated China's public health reform, and precisely at that time the importance of EBPH was realized by scholars. By applying the principles and methods of evidence-based medicine, the Chinese Cochrane Center specifically developed evidence-based strategies for preventing and addressing unexpected public health events. After the “Wenchuan earthquake” in Sichuan Province in 2008, more efforts concerning evidence-based strategies were made, involving emergency management, modes of rescue, physical and mental interventions, rehabilitation, finance, and logistics [17]. After 2008, the scope of EBPH research in China gradually expanded to wide public health domains, such as epidemiology, health economics, and health management, all of which aim at making decisions on the basis of the best available scientific evidence [18]. The existing research in China has mostly focused on developing or improving evidence-based measures or approaches. However, in regard to how to translate evidence-based science into practice, studies of current EBPH utilization and its impeding factors are scarce.

Consequently, the main objective of the study is to review and assess the current EBPH implementation status and explore its barriers in China. As an exploratory study, we chose Shanghai as the sample region because EBPH is new to China and we seek to investigate the situation in this most developed region to reveal the overall situation in China. The findings may also provide effective suggestions for low- and middle-income countries that are considering the introduction and practice of EBPH in the public sector to promote their EBPH implementation.

## 2. Methods

### 2.1. Analytical Framework

To analyze the status and major determinants of EBPH practices in China, we formulated an interview instrument based on Damschroder et al.'s Consolidated Framework for Implementation Research (CFIR) [19], which has been widely used by prior studies addressing health innovation implementation [20–22]. The CFIR provides a systematic overarching typology to promote the development of implementation theory and verification of the target and scope of EBPH. It comprises the following five major domains: intervention characteristics, inner setting, outer setting, individuals, and implementation process (Table 1). The subcategories or constructs are also displayed in Table 1. In summary, the framework indicates that the obstacles to EBPH practices can essentially be reflected in different domains and constructs. By using the CFIR framework, we developed interview instruments for data collection in healthcare institutions. The interview instrument is available on request, and the brief sample questions are listed in Table 1.

### 2.2. Data Collection

*In our study, the participants consisted of 14 *officials from the Chinese Centers for Disease Control and Prevention (CDC) and 82 practitioners from 10 community healthcare centers, 8 secondary hospitals, and 6 tertiary hospitals during the period of June 2015 to October 2015. We included secondary and tertiary hospitals because they also undertook much of the public health work in China. Ultimately, as an exploratory study, this study can reflect the most advanced level of EBPH implementation in China.

For all the institutions we visited during our field study, we do not use their names in our analysis below due to privacy concerns. Instead, we refer to them as Institution A1, A2,…, A10 (A represents community healthcare centers), B1, B2,…, B8 (B represents secondary hospitals), and C1, C2,…, C6 (C represents tertiary hospitals). The CDC is assigned the label of D1.

### 2.3. Data Analysis

In this study, data were analyzed for thematic content guided by the CFIR framework, and the indicative coding was obtained by iterative methods. We then identified key issues and problems in the implementation of EBPH in Shanghai, China. Additionally, attempting to develop a clear understanding of EBPH practices and their impediments in China, we collected and analyzed the literature from the Chinese National Knowledge Infrastructure Database and Wan Fang Database (the two largest databases in China), and we related policies issued by the government. All these data were collected from January 1, 2000, to March 31, 2016.

## 3. Results

### 3.1. Status of EBPH Practices in Shanghai

Overall, we found that a growing emphasis has been placed on public health in recent years in China. For instance, prompted by the national health bureau's request that basic public health programs be conducted, the Shanghai health bureau created its key healthcare list and launched a public health campaign beginning in 2011 [23]. These programs referred to 12 categories, including disease control, vaccination, child healthcare, maternal healthcare, gerontology, and health education. These activities generally involved EBPH practices. Unfortunately, however, the condition of practice in Shanghai remains unsatisfactory.

At the public health practitioners' level, on the whole, it was found that health practitioners knew and practiced more evidence-based medicine but clearly less evidence-based public health.* “The evidence we use most often is that about the application of medication*…* For population intervention, personally, I'm not very familiar with that or how to do it”* (A3-3). Furthermore, by comparing the status of EBPH practices in community healthcare institutions with that in higher-grade ones, we found that interviewees in higher-grade institutions had more access to EBPH. The discrepancy lay in the higher-grade institutions' access to more academic projects. Due to the lack of funding, EBPH practices were mainly initiated in the form of academic projects. The scope of the implementation depended on the project, such as the population and regions included and the budget dedicated. Moreover, it was noteworthy that although many public health practitioners had implemented evidence-based practices in their work because they had not received systematic and orthodox training on EBPH, they applied little EBPH.

At the CDC officials' level, we were disappointed to find that among the 14 interviewees four senior officials were not even familiar with the concept of EBPH. Others knew a bit but had not recognized its importance and significance. In fact, their work was highly related to EBPH because they must take measures based on the upper government's guidance, such as the policy of “Tertiary Prevention” targeting chronic disease prevention and control. Overall, they seemed to be passive policy receivers but not proactive in proposing or improving new approaches/policies based on accumulated data or experience.

### 3.2. Impediments to EBPH Implementation

#### 3.2.1. Intervention Characteristics

Among the 82 interviewees from healthcare institutions, most answered with external channels, such as published literature, guidelines, policies, seminars, and academic conferences. Moreover, practitioners in community healthcare centers deemed it more difficult to obtain good-quality intervention sources. Indeed, most of the database of published literature was not free of charge, and it was a great burden for fundamental institutions to afford.

#### 3.2.2. Inner Setting Factors

The inner setting comprises the spectrum of organization-level strategies that have the potential to influence EBPH implementation. In this study, the interviewees expressed that there were many institutional inhibitors. According to the CFIR framework, the inner setting factors comprise internal structural characteristics, networks and communications, implementation climate, and leadership engagement [20]. In fact, these internal constructs are correlated with each other.

*(1) Structural Characteristics*. Interviewees in this study stated that to better promote EBPH practices, “*a team with strong ability in all aspects is required*” (C1-1). However, extracted results showed that although inner hierarchical structure was reasonable, implementation was restricted by the capacity of both the leaders and the subordinates, reflecting their lack of capacities to cooperate and operate the given programs by observing the mechanism instead of practicing it spontaneously.

*(2) Networks and Communications.* Another important issue is how we can effectively transmit our ideas or experiences to others. As one practitioner noted, “*everyone's part in the organization is written in detail and should be very clear*…* Also, the bridge between the doctors and the management level is important, and it's quite good in our organization*” (A3-2).

*(3) Implementation Climate*. In our analysis, we found that among the diverse climate categories, the lack of organizational incentives was mentioned most frequently. Extracted information indicated that until now there had been no direct incentive from the government in the form of funds to encourage public health practitioners to apply EBPH-related programs, although some were encouraged by the government. Instead, the most common way to practice EBPH was in the form of academic projects. When asked “how much support do you feel the department provides for the processes necessary for utilizing evidence-based programs/policies?” interviewees answered “*we have our own research*” (A1-1) and “*the support is project-based, like the project approved by the Shanghai Municipal Commission of Health and Family Planning, or by our hospital*” (C1-4). In this case, the duration of the funding depended on the term of the project.

*(4) Leadership Engagement.* In China, executive leaders in healthcare institutions are appointed administratively by the health bureau. Additionally, an interesting and common phenomenon is that even institutions are endowed with certain autonomy; if not forced by the upper government, executives are unwilling to take measures because their effects may not be known during their tenure [24]. Therefore, new technologies or approaches cannot be utilized and promoted timely. As stated by one physician in C2: “*The same is true with those officials who are willing to make their own achievements. They manage to hold to a point and make it his/her achievement, and leave over other things. For example, one official worked on this, but the new leader changed ideas and changed everything. This is common in China.*”

Currently, because an academic project is the conventional way to implement EBPH, many public health practitioners desire their administrators to provide more such opportunities. As stated by a physician in a community hospital, “*I do not know what the chronic disease management programs in other community-based health services centers look like, ours used to be a mess. But after the new dean got here, our programs become sound*” (A3-2).

#### 3.2.3. Outer Setting Factors

Generally, the outer setting comprises the economic, political, and social context within which an organization resides. Normally, the implementation of a new approach will be influenced by outer setting factors mediated through changes in the inner setting [25].

*(1) External Policies and Incentives.* Among the broad constructs that encompass external factors to spread interventions, the policies, regulations, guidelines, and public or benchmark reporting count for a great deal [26], in this study, when asked “can you think of any external condition that can help you to implement the evidence-based public health interventions?” impressively, among the 82 practitioners, 53 noted that microlevel policies were the most important and urgent measures. Related problems with the policy can be manifested as lack of funding and execution power. Practitioners noted that “*encouraging young and promising doctors to do EBPH work in community-based health centers requires money; more funding should be provided by the government*” (A2-2). Concerning the power to execute current policy, some interviewees said that although the policy of “classification and treatment of constipation among different health institutions” was advocated because the referral medical mechanism did not function well, patients with constipation usually went directly to the higher-grade hospital and it was difficult for the community health centers to track them and follow up.

Notably, the third-party-neighbourhood committees in China's administrative system are helpful in promoting the public health. Although they are not health institutions, their role of communication and organization between health institutions and the public facilitates public health work, which is a typical phenomenon in China. The neighbourhood committee is the basic autonomous organization of self-management, self-supervision, and self-service, and each community has its own neighbourhood committee. Typically, when new policies are issued by the government, this type of committee is also asked by the government to offer assistance to other targeted institutions. In this case, it can help the public realize and accept health practitioners' EBPH practices and facilitate their work.

*(2) Patient Needs and Resources. *Patients are the direct recipients of health programs; conversely, their feedback or requirements can be beneficial for health practitioners. If practitioners can extract experience and apply it to new practice, doing so is obviously a good EBPH practice. In reality, interviewees in this study reflected that this practice was weak, especially in the community health centers. The key problem lay in low patient compliance. One physician stated, “*we always provide the patients with appropriate guidance or treatment according to the guidelines, such as coronary heart disease and hyperlipemia. However, many of the patients' compliance is not good. Because health management requires cooperation between the physicians and patients, a lack of this will further result in low EBPH practice*” (B1-1).

#### 3.2.4. Individual Factors

Greenhalgh et al. (2004) described the significant role of individuals as carriers of cultural, organizational, professional, and individual mindsets, norms, interests, and affiliations. Individuals make choices and can wield power and influence over others with predictable or unpredictable consequences for implementation [27].

In this study, such individual factors were reflected as lack of time and energy to think and participate, ineffective ability to conduct the program, and lack of awareness of EBPH. By comparison, it was shown that feedback from community healthcare centers was more related to limited skill and lack of consideration, while general hospitals reflected that they failed to practice EBPH due to high workloads and limited time. Another interesting phenomenon among the Chinese public health practitioners was that although some had conducted EBPH programs, they failed to identify EBPH practices. This low consciousness was directly caused by a lack of professional training.

#### 3.2.5. Implementation Process

According to the CFIR framework, the implementation process contains five essential activities that range across organizational change models: planning, engaging, executing, reflecting, and evaluating [20]. Through analysis, we found that there was no clear EBPH implementation process for those institutions that already practiced EBPH in Shanghai. One interviewee stated, “*we are doing research in our communities or we are working towards this*” (A3-1), signifying a lack of systematic implementation process. However, establishing a regular EBPH process is not as easy as it sounds because it requires department management, quality management, evaluation, and improvement.

In summary, the CFIR's overarching structure supports the exploration of essential factors that may be encountered during EBPH implementation in Shanghai. A brief summary of our interviews is presented in Table 2.

## 4. Discussion

### 4.1. Key Challenges of EBPH Implementation in China

Regarding the five CFIR domains, evidence, the institutional system, government support, and individuals' awareness persist as the key challenges to implementing EBPH.

#### 4.1.1. Insufficient and Low-Quality Public Health Evidence in China

The primary problem with public health evidence in China lies in its insufficiency and low quality. Comparatively, developed countries have accumulated persuasive national guidelines and policies in these aspects. In the US, the national level of evidence, such as the Guide to Community Preventive Services, has been put into practice [28]. In Australia, “Health-Evidence.org” is a widely accepted evidence source. Canada has established an extensive online set of evidence reviews and resources for scholars or practitioners to disseminate EBPH (http://www.healthevidence.org/) [29, 30]. Additionally, the Cochrane Collaboration provides an abundant repository in many developed countries [31]. The results showed that Chinese public health professionals have a pivotal position in efforts to change the current circumstances.

The change requires the collective efforts of both the external government and internal healthcare institutions. For the government, construction of a decision-making database that suits Chinese people should be advanced, and both the CDC and other public health administrations must utilize population data. For institutions, the priority should be providing more opportunities for their employees to obtain evidence, such as internal training and external academic meetings.

#### 4.1.2. Irrational Institutional System without Effective Communication and Incentive Mechanisms

Qualitative results indicated that there were few effective communication systems for EBPH practice within institutions. Additionally, there was a lack of sufficient incentives for practitioners to promote EBPH while they are already busy with work. All these factors made it disadvantageous for public health practitioners to acquire and spread evidence-based philosophy and methods. Another vital obstacle was the irrational financial incentive mechanism. The extracted information showed that the initiation of EBPH was based on academic projects, making implementation restricted by the duration and amount of the funding.

To establish favourable mechanisms, much should be considered involving intensified training and other communication methods. Furthermore, multiple incentives such as performance reviews, promotions, and less tangible incentives like increased stature or respect should be explored.

#### 4.1.3. Lack of Resources for EBPH Practices due to Limited Government Support

Although the government health agencies aimed to achieve universal public health program coverage in China, the coverage rate remained far from the established standards [32]. As revealed, one important reason was that these projects incurred expenses far beyond their budget. As a result, institutions and practitioners claimed they could not sustain the projects. Another reason was the government's lack of EBPH consciousness, with the results showing that some public health officials did not even understand EBPH.

Except for more direct invested funds, other government support should be exploited. For instance, government health agencies can provide more public health-related projects to induce practitioners or institutions to focus on public health. To increase health officials' awareness, much support should be provided to improve their innovation and scientific decision-making abilities.

#### 4.1.4. Public Health Practitioners' Weak Awareness of EBPH Practices in China

EBPH is a relatively new ideology in China. Although it has been recognized and applied in public health practitioners' daily work to some degree in Shanghai, we found that many practitioners failed to realize its essence and potential advantages due to their conventional awareness and work patterns.

Under the current limitations in internal and external incentives, more active approaches should be explored to inspire individuals. Personal presentations and institution-based training (e.g., information retrieval, EBPH methodology popularization) are good methods that can assist individuals to achieve a broad reach. Additionally, because practitioners in higher-grade health institutions in Shanghai are more aware of and practice more EBPH, policies should be made to encourage the workforces in community healthcare centers, who are also the main force of future public health according to China's strategic health policy, to better stimulate EBPH practice.

### 4.2. International Comparison of EBPH Practices

The necessity of using EBPH to improve public health practice is well recognized by practitioners and researchers [2, 4, 6]. However, we found developing countries seriously delayed in this region. Numerous studies have shown that in developed countries, such as the US and many European countries, scholars are focusing on disseminating science to exploit effective interventions or strategies to elevate the quality and efficiency of evidence-based public health programs [2, 16, 33]. For instance, Allen et al.'s (2013) research designed multiphase dissemination approaches that included multiday in-person training workshops, electronic information exchange modalities, and remote technical assistance. Yost et al. noted that in Canada the National Collaborating Center for Methods and Tools (NCCMT) has established an online, searchable, and freely accessible collection of methods and tools to support knowledge translation in public health [34].

However, in China and most developing regions, existing research on EBPH is still in its early stages and exploratory studies of current status and its influencing factors are scarce, as is implementation science [35, 36]. Scattered studies mainly address the acquisition of health-related evidence. By exploring the current status of and impediments to EBPH in China, this study can provide inspiration for EBPH implementation and public health promotion in other developing countries and regions that suffer from the same limitations.

## 5. Limitations

Several limitations of the current review should be noted. First, the data were self-reported, and we had no ability to objectively compare the reported activities with actual practices. Second, a convenience sample of key informants limits the generalizability of our study but was an appropriate approach in this exploratory study. Additionally, we could not avoid social desirability because a potential source of bias existed. Third, the international literature may not be able to convey the panorama of EBPH studies in other developing regions; however, restricted by language, we cannot obtain the local literature. Fourth, this study only depicted the present situation in Shanghai; however, Shanghai's experiences with EBPH are likely not generalizable to other areas in China because Shanghai is unique in its high education and income and access to international resources. However, because EBPH is new to China, we presume that healthcare providers in Shanghai have more opportunities to obtain evidence-based public health knowledge and practice it for us to acquire the up-to-date situation of China. We realized that much still remains to be explored in other regions of China.

## 6. Conclusions

In summary, by applying the CFIR theory, which accurately describes the overarching contexts of the obstructive constructs qualitatively, we determined that China lags in the cognition and utilization of EBPH and that the operation of EBPH is primarily project-based. Considering the key challenges of EBPH promotion in China, it seems more feasible for healthcare institutions to take the initiative to explore multiple methods to facilitate and improve the development of EBPH. This study will become the baseline information on EBPH evaluation in China, and we hope it will be beneficial for other developing regions in launching and deepening their research and practice.

## Figures and Tables

**Table 1:** The Consolidated Framework for Implementation Research.

Domain	Constructs	Questions and examples
Intervention characteristics	Intervention source, evidence strength and quality, relative advantage, adaptability, trialability, complexity, design quality and packaging, cost	How would you describe the current evidence-based interventions?
Inner setting	Structural characteristics, networks and communications, culture, implementation climate, leadership engagement	Can you identify any organizational barriers that impede your ability to implement EBPH?
Outer setting	External policies and incentives, patient needs and resources, cosmopolitanism, peer pressure	Can you identify any external factors that impede your ability to implement EBPH?
Individual characteristics	Knowledge and beliefs about the intervention, self-efficacy, individual stage of change, individual identification with organization, other personal attributes	Can you identify any personal barriers that impede your ability to implement EBPH?
Implementation process	Planning, engaging, executing, reflecting and evaluating	How would you describe the situation of your department as it relates to implementing evidence-based processes?

*Note: *the framework was cited from [19].

**Table 2:** CFIR perspectives on EBPH implementation issues.

CFIR dimension	Key issues and problems
Intervention characteristics	Intervention sources were limited, and most were from external channels; the quality of the evidence was not ideal.
Inner setting	The implementation was restricted by the capacity of both the leaders and the subordinates; there was a lack of organizational incentives and rewards for EBPH implementation.
Outer setting	Although external policies proposed that practitioners implement evidence-based practices, the execution of the policies was not good, and the government funding was insufficient; there was low patient compliance with the practitioners' EBPH practices.
Individual characteristics	There was a lack of time and energy to practice EBPH and low awareness and identification of EBPH.
Implementation process	There was no clear process for EBPH implementation.

## Abbreviations

EBPH: Evidence-based public health
CDC: Centers for Disease Control and Prevention
CFIR: Consolidated Framework for Implementation Research.
